# Mechanisms underlying ICU muscle wasting and effects of passive mechanical loading

**DOI:** 10.1186/cc11841

**Published:** 2012-10-26

**Authors:** Monica Llano-Diez, Guillaume Renaud, Magnus Andersson, Humberto Gonzales Marrero, Nicola Cacciani, Henrik Engquist, Rebeca Corpeño, Konstantin Artemenko, Jonas Bergquist, Lars Larsson

**Affiliations:** 1 Department of Neuroscience, Clinical Neurophysiology, Uppsala University, Entrance 85, Uppsala University Hospital, Uppsala 751 85, Sweden; 2 Department of Neuroscience, Neurosurgery, Uppsala University, Entrance 85 Uppsala University Hospital, Uppsala 751 85, Sweden; 3 Department of Chemistry - Biomedical Center, Uppsala University, Husargatan 3 (D5), Uppsala 751 24, Sweden

## Abstract

**Introduction:**

Critically ill ICU patients commonly develop severe muscle wasting and impaired muscle function, leading to delayed recovery, with subsequent increased morbidity and financial costs, and decreased quality of life for survivors. Critical illness myopathy (CIM) is a frequently observed neuromuscular disorder in ICU patients. Sepsis, systemic corticosteroid hormone treatment and post-synaptic neuromuscular blockade have been forwarded as the dominating triggering factors. Recent experimental results from our group using a unique experimental rat ICU model show that the mechanical silencing associated with CIM is the primary triggering factor. This study aims to unravel the mechanisms underlying CIM, and to evaluate the effects of a specific intervention aiming at reducing mechanical silencing in sedated and mechanically ventilated ICU patients.

**Methods:**

Muscle gene/protein expression, post-translational modifications (PTMs), muscle membrane excitability, muscle mass measurements, and contractile properties at the single muscle fiber level were explored in seven deeply sedated and mechanically ventilated ICU patients (not exposed to systemic corticosteroid hormone treatment, post-synaptic neuromuscular blockade or sepsis) subjected to unilateral passive mechanical loading for 10 hours per day (2.5 hours, four times) for 9 ± 1 days.

**Results:**

These patients developed a phenotype considered pathognomonic of CIM; that is, severe muscle wasting and a preferential myosin loss (*P *< 0.001). In addition, myosin PTMs specific to the ICU condition were observed in parallel with an increased sarcolemmal expression and cytoplasmic translocation of neuronal nitric oxide synthase. Passive mechanical loading for 9 ± 1 days resulted in a 35% higher specific force (*P *< 0.001) compared with the unloaded leg, although it was not sufficient to prevent the loss of muscle mass.

**Conclusion:**

Mechanical silencing is suggested to be a primary mechanism underlying CIM; that is, triggering the myosin loss, muscle wasting and myosin PTMs. The higher neuronal nitric oxide synthase expression found in the ICU patients and its cytoplasmic translocation are forwarded as a probable mechanism underlying these modifications. The positive effect of passive loading on muscle fiber function strongly supports the importance of early physical therapy and mobilization in deeply sedated and mechanically ventilated ICU patients.

## Introduction

The mortality rate has typically been the primary outcome considered in reports on critically ill ICU patients [1,2]. Recent advances in ICU care have significantly improved the survival rate of critically ill ICU patients. However, modern critical care also needs to address post-ICU complications and long-term quality of life. Several recent studies have reported a severely impaired quality of life in critical illness survivors several years after ICU and hospital discharge, primarily due to an impaired neuromuscular function [1,3,4].

During ICU treatment, a large proportion of the critically ill, mechanically ventilated ICU patients develop severe muscle wasting and weakness of limb muscles due to acquired myopathy, neuropathy or a combination of both. Acquired myopathy is by far the most common cause underlying this specific muscle weakness, but is frequently misdiagnosed due to insufficient diagnostic criteria or methodological limitations [5,6]. This myopathy, characterized by reduced muscle membrane excitability and a preferential loss of the molecular motor protein myosin, has been given multiple different names, the most common being acute quadriplegic myopathy or critical illness myopathy (CIM). Primary disease, sepsis and multiorgan failure undoubtedly contribute to the impaired muscle function, but there is heterogeneity of underlying disease and pharmacological treatment among patients with similar outcomes. The common components of ICU treatment *per se *- such as bed rest, muscle unloading, mechanical ventilation, and sedation - are thus probably all directly involved in the progressive impairment of muscle function during long-term ICU treatment.

Using a unique experimental ICU model allowing detailed studies of skeletal muscle in mechanically ventilated, deeply sedated, pharmacologically paralyzed and extensively monitored rats for several weeks [7], we have recently shown that the complete mechanical silencing associated with the ICU condition (absence of external strain related to weight-bearing, and internal strain in the muscle fiber caused by myosin-actin activation) induces a phenotype identical to the acquired myopathy in ICU patients with CIM [8]. Mechanical silencing has accordingly been forwarded as an important etiological factor underlying this specific myopathy [8].

The ability of the muscle cell to sense, process, and respond to mechanical stimuli is an important regulator of gene expression and protein synthesis and is therefore an important regulator of physiological and pathophysiological function, an interplay sometimes referred to as tensegrity [9-13]. In a clinical study, Griffiths and coworkers demonstrated that unilateral continuous passive movement for 3 hours three times per day during 7 days preserved the architecture of the muscle fiber and protein loss in five mechanically ventilated, pharmacologically paralyzed and critically ill ICU patients [14]. Furthermore, a number of different recent studies have shown that early intense physical therapy in ICU patients significantly shortens the ICU and hospital stays, reduces healthcare costs and improves overall patient quality of life [15-18]. These encouraging results have the potential to induce a paradigm shift in attitudes towards physiotherapy and the prevention of ICU muscle wasting and weakness. However, the mechanisms underlying intervention effects on skeletal muscle structure and function in immobilized ICU patients remain unknown.

This study aims to unravel the mechanisms underlying the muscle atrophy seen in deeply sedated and mechanically ventilated ICU patients and how these mechanisms can be affected by passive mechanical loading. Muscle gene/protein expression, post-translational contractile protein modifications, muscle membrane excitability and regulation of muscle contraction at the cellular level were explored after unilateral passive standardized mechanical loading of ankle joint flexors and extensors for 10 hours per day (2.5 hours, four times per day) for 9 ± 1 days (7 to 11 days). The cross-sectional area (CSA) of the tibialis anterior (TA) muscle and electrophysiological properties of the muscle and peripheral motor nerves were monitored during the observation period. At the end of the observation period, percutaneous muscle biopsies were taken from both the loaded and unloaded TA muscle and were analyzed for gene/protein expression, post-translational modifications (PTMs) and regulation of muscle contraction at the single muscle cell level.

The purpose of this study was to better understand the mechanisms underlying CIM, and to evaluate the effects of passive mechanical loading on muscle structure and function in sedated and mechanically ventilated ICU patients. We hypothesized that mechanical silencing is a dominant factor triggering the muscle wasting and weakness associated with CIM in ICU patients, and that the passive mechanical loading alleviates the muscle wasting, the preferential myosin loss and the loss in specific force (maximum force normalized to muscle fiber CSA). The results from this study confirm that the ICU intervention *per se *(immobilization, sedation and mechanical ventilation) plays a critical role in the preferential myosin loss, a major diagnostic feature of CIM

## Materials and methods

### Patients and control subjects

A total of seven mechanically ventilated ICU patients (four females and three males, aged 56 to 67 years) numbered M1 to M7 were included in this study. The clinical history and medications used by each patient are summarized in Table 1. Patients anticipated to require mechanical ventilation for 10 consecutive days or longer were recruited. Patients had typically been exposed to mechanical ventilation and immobilization for 0 to 3 days (1.7 ± 0.9 days) prior to initiating the intervention and monitoring due to delays related to obtaining signed informed consents. Patients with a previous history of neuromuscular disease were not included in the study. There was no evidence of severe sepsis (sepsis with organ dysfunction) in any of the patients according to the 2001 SCCM/ESIMC/ACCP/ATS/SIS International Sepsis Definitions Conference. None of the patients received systemic administration of neuromuscular blocking agents, and only one patient (M1) received administration of inhaled corticosteroids (daily dose: 1 mg) due to asthma. Propofol was intravenously administered in all patients.

**Table 1:** Clinical history and medications used by each ICU patient

Patient	Age (years)	Gender	Start respirator before study (days)	Biopsy time (days)^a^	Primary disease	History	Pharmacology	SAPS II
M1	56	F	2	8	Intraventricular hemorrhage	Whiplash, status asthmaticus, COPD	Corticosteroids, antibiotics	37
M2	62	M	2	9	Acute subdural hematoma	Hypertony, alcoholic	Antibiotics	50
M3	67	M	2	8	Subarachnoid hemorrhage	Hypertony	Antibiotics	41
M4	62	F	3	9	Intracerebral hemorrhage (left)	Fibromyalgia, hypertony, hypothyroidism	Antibiotics	41
M5	63	F	0	9	Intraventricular hemorrhage	Diabetes, obesity, hypertony, COPD	Antibiotics	39
M6	59	F	1	11	Cerebellar hemorrhage		Antibiotics	34
M7	62	M	2	7	Bilateral cerebellar infarction		Antibiotics	42

COPD, chronic obstructive pulmonary disease; F, female; M, male; SAPS, Simplified Acute Physiology Score. ^a^After the intervention started.

Written informed consent was obtained from patients' close relatives prior to beginning the study. This study was approved by the local Ethical Committee on Human Research at Uppsala University Hospital, Uppsala, Sweden.

Muscle biopsies were obtained from the TA muscle on both the unloaded and loaded sides using the percutaneous conchotome method on the final day of the observation period. Muscle biopsies were not taken at the start of the intervention in order to eliminate the risk of the first biopsy sampling procedure interfering with the results from the biopsy obtained at the end of the observation period. Each biopsy was dissected and treated as previously described [19]. For details, please see Additional file 1.

Muscle biopsies from the TA muscle from a total of eight healthy control subjects (seven females and one male, age 67 to 78 years) were included for quantitative RT-PCR and myosin:actin protein ratio comparisons. All muscle samples from patients and controls were obtained. A total of 40 healthy control male and female subjects (aged 23 to 75 years) were included as reference material for the comparison of the compound muscle action potential (CMAP) amplitude during direct and indirect (nervus fibularis) TA muscle stimulation.

For comparison of mass-spectrometry protein PTMs, 13 healthy male control subjects (aged 25 to 89 years) were included for analysis of type I and type IIa myosin heavy chain (MyHC) isoforms from limb muscles (vastus lateralis).

### Mechanical loading

Ankle joint flexors and extensors were passively loaded for 2.5 hours four times per day during 7 to 11 days (9 ± 1 days) using a Kinetec™ Breva™ Ankle CMP machine (A Patterson Medical Company, Tournes, France); that is, continuous passive anatomically correct motion from 30° plantar flexion to 25° dorsiflexion was generated at a speed corresponding to 150°/minute.

### Ultrasound measurements

The left and right TA CSAs were measured every day during the intervention period (7 to 11 days) using a real-time ultrasound scanner (Siemens Acuson Antares Ultrasound System, Mountain View, CA, USA) with a 9 to 4 MHz linear array transducer. The principles of ultrasound scanning have been described previously [20].

Scans were taken transversally on relaxed muscles at three locations: 50%, 40% and 30% of the distance from the proximal part of the fibula head to the distal tip of the lateral malleolus. These distances were marked on the skin with a marker pen to eliminate intra-individual variations in the location of measurements during the observation period. Ultrasound coupling gel (Polaris II; GE Medical Systems, Aulnay sous Bois, France) was applied to the skin and transducer head. The transducer was placed at the different locations and held perpendicular to the skin to ensure a clear image and perpendicular to the direction of TA muscle to acquire transverse measurements. The captured muscle images were stored, the region of interest (TA muscle mass without bone and fascia) was manually selected and the CSA was measured using a SieScape™ panoramic imaging processor (Siemens AG, Erlangen, Germany).

The mean CSA was calculated as the mean of three consecutive measurements at the three different locations (50%, 40% and 30%) on each leg. Coefficients of variations were calculated from the two initial CSA measurements at each location and from the average of all three locations combined. Coefficients of variation were <5% for all measurements and on average were 1.77% for measurement at each single location and 1.04% when taking the mean of the three different locations.

### Electrophysiological measurements

Motor (nervus fibularis, and tibialis) and sensory (nervus suralis and fibularis superficialis) nerves were measured bilaterally (Keypoint Medtronic, Skovlunde, Denmark) using a surface electrode both for stimulation and recording. Studies were performed on the first and final days of the observation period in all patients, and two of the seven patients (M6 and M7) were monitored every second day during the observation period. Nerve conduction velocities were compared with reference values from age-matched and height-matched control subjects (Department of Clinical Neurophysiology, Uppsala University Hospital). The CMAP amplitudes upon supramaximal motor nerve stimulation were measured from the musculus extensor digitorum brevis (nervus fibularis stimulation) and musculus abductor hallucis (nervus tibilalis stimulation). On the final day of the period, concentric needle electromyography was performed in the musculus vastus lateralis and tibialis anterior bilaterally (Keypoint Medtronic).

The CMAP amplitude was measured upon direct supramaximal musculus tibialis anterior stimulation (dmCMAP) and compared with the CMAP amplitude in response to supramaximal nervus fibularis stimulation (neCMAP) bilaterally on the final day of the observation period. CMAP amplitudes were measured peak to peak. neCMAP was measured in response to supramaximal stimulation of nervus fibularis at the level of the fibular head and recording electrodes were kept in the same position as during the dmCMAP measurement. The neCMAP:dmCMAP amplitude ratio was calculated. The limb skin temperature was kept at >32°C. Filter settings were 2 Hz to 5 KHz and the stimulus duration was 0.1 ms (1 Hz stimulation rate). For details, please see Additional file 1.

### Contractile measurements of single muscle fibers

Single muscle fiber experiments were performed as described previously [8]. In brief, a fiber segment 1 to 2 mm long was attached to a force transducer (model 400 A; Aurora Scientific, Aurora, ON, Canada) and a lever arm system (model 308B; Aurora Scientific). While the fiber segments were in relaxing solution, the sarcomere length was set at 2.65 to 2.75 μm by adjusting the overall segment length [21] and the resting tension was assessed. The fiber was then moved to activating solution (pCa 4.5) in which all the recordings were performed. The focusing control of the microscope was used as a micrometer. Fiber CSA was calculated from the diameter and depth, assuming an elliptical circumference, and was corrected for the 20% swelling that is known to occur during skinning [22]. Because single muscle fibers expressing the type II MyHC isoform were absent in several patients, CSA and force measurements were restricted to muscle fibers expressing the type I MyHC isoform.

### Enzyme histochemistry and immunocytochemistry

Cross-sections (10 μm) were cut perpendicular to the longitudinal axis of muscle fibers with a cryostat (2800 Frigocut E; Reichert-Jung GmBH, Heidelberg, Germany) at -20°C. The sections were stained for NADH (3.2 mg Nicotinamide adenine dinucleotide, 8.0 mg Nitro blue tetrazolium, 2.0 ml 3-(N-Morpholino)propanesulfonic acid solution, 8.0 ml distilled H_2_O).

Muscle fiber CSA measurements were restricted to type I fibers since type II fibers were absent in some of the patients. Type I fiber CSA was measured for 50 fibers in the central region of the biopsy cross-section and measured using an inverted microscope (Axiovert 40 CFL; Carl Zeiss, Jena, Germany) and imaging software (Compix Simple PCI 6; Compix Inc., Sewickley, PA, USA).

Neuronal nitric oxide synthase (nNOS) expression was assessed on 10 μm TA cryo-sections from patients and healthy controls. Sections were blocked using Background sniper (Histolab, Göteborg, Sweden).

Primary antibodies were rabbit anti-nNOS (Invitrogen, Carlsbad, CA, USA) and rat anti-Laminin gamma 1 (Millipore, Billerica, MA, USA). Secondary antibodies were anti-rabbit Cy3 donkey and anti-rat Dylight 488 goat conjugates (BioLegend, San Diego, CA, USA). Nuclei were visualized with 4',6-diamidino-2-phenylindole. All samples were stained with identical primary and secondary antibody dilutions and immunofluorescence was analyzed by confocal microscopy (LSM510 Meta; Zeiss).

### Myosin:actin protein ratio

TA 10-μm cryo-sections were dissolved in 100 μl urea buffer (8 M; 120 g urea, 38 g thiourea, 70 ml H_2_O, 25 g mixed bed resin, 2.89 dithiothreitol, 1.51 g Trizma base, 7.5 g SDS) after centrifugation and heating (90°C for 2 minutes). The total protein content of the samples was measured with Pierce^® ^660 Protein assay (ThermoFisher Scientific Inc., Rockford, IL, USA) according to the manufacturer's instructions. The samples were run on 12% SDS-PAGE and gel bands corresponding to actin and myosin were identified and quantified as previously described [8]. These values were used to determine myosin:actin protein ratios.

### Post-translational modifications

Cross-sections from TA from ICU patients and vastus lateralis from controls were run on 6% SDS-PAGE gel. Gel bands corresponded to myosin heavy chain I and IIa. Samples were digested in gel, separated with 40-minute gradient RP-nanoHPLC and analyzed online using a 7-Tesla LTQ-FT Ultra tandem mass spectrometer (ThermoFisher Scientific Inc.) modified with a nano electrospray ion source (ProxeonBiosystems, ThermoFisher Scientific Inc.). A high-resolution survey scan followed by low-resolved mass spectrometry/mass spectrometry scans of the five most abundant peaks were used. Peptide identification was performed using the Mascot search engine, allowing two missed cleavages and a set of variable post-translational modifications (that is, multiple oxidations, methylations, and phosphorylations). The myosin modeling used in the study has been described extensively elsewhere [23] and was visualized with UCSF Chimera [24]. For detailed information, please see Additional file 1.

### Quantitative real-time PCR

Quantitative RT-PCR was used to quantify the mRNA levels for human type I MyHC, type IIa MyHC, skeletal α-actin, myosin binding protein (MyBP)-C_slow _and MyBP-H ([GenBank:M58018], [GenBank:AF111784], [GenBank:NM_001100], [GenBank:NM_002465] and [GenBank:NM_004997], respectively).

Total RNA was extracted from frozen TA samples and then quantified as previously described [5]. Then 100 ng total RNA from TA samples were reverse transcribed to cDNA using Qscript cDNA supermix (Quanta Biosciences, Gaithersburg, MD, USA). cDNA was amplified in triplicate using the MyiQ™ single-color real-time PCR detection system (Bio-Rad Laboratories, Inc., Hercules, CA, USA). For details regarding the PCR protocol and primers used, please refer to Additional file 1.

### Statistical analysis

SigmaPlot software (Systat Software, Inc., San Jose, CA, USA) was used to generate descriptive statistics. Means, standard errors of the means and linear regression analysis were calculated according to standard procedures. A paired *t *test was used in pairwise comparisons between unloaded and loaded legs. One-way analysis of variance and the Tukey *post-hoc *test were used when comparing multiple groups. When the normality test failed, a one-way analysis of variance on ranks (that is, Kruskal-Wallis test) and the Dunn's *post-hoc *test were performed. Differences were considered significant at *P *< 0.05.

## Results

### ICU patients

This study was carried out in seven mechanically ventilated ICU patients between 7 and 11 days (9 ± 1 days). All subjects were adults (62 ± 1 years). Patient characteristics are summarized in Table 1 along with their primary diagnosis, medications used, biopsy time and days of exposure to mechanical ventilation prior to starting the intervention. None of the patients had a history of neuromuscular disorders. No spontaneous movements in the deeply sedated patients were recorded during the observation period. Average body weight decreased (*P *< 0.05) from 79.9 ± 5.5 to 78.3 ± 5.4 kg at the end of the observation period.

### Ultrasound measurements

**Figure 1:**
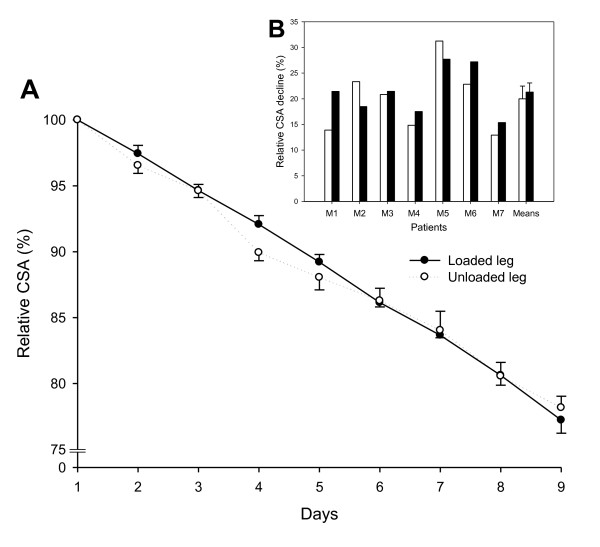
**Ultrasound measurements of tibialis anterior cross-sectional area**. **(A**) Relative cross-sectional area (CSA) during the intervention period (9 ± 1 days). Solid line, loaded side; dashed line, unloaded side. Values are mean ± standard error of the mean (SEM). The value at day 1 is equivalent to 100%. **(B) **Relative CSA decline in the loaded leg (black bars) and the unloaded leg (white bars) for each ICU patient on the final day of the intervention, and mean ± SEM for all patients.

A linear decline was observed in TA CSA during the observation period on both the loaded side (*r*^2 ^= 0.999, *P *< 0.001) and the unloaded side (*r*^2 ^= 0.991, *P *< 0.001) (Figure 1A). On the final day of the observation period, TA CSA had declined by 21 ± 1% and there was no difference in TA CSA between the loaded and unloaded legs (Figure 1B). The results thus demonstrate a decline in TA CSA proportional to the length of the ICU stay, but mechanical loading did not influence the overall TA muscle CSA.

### Electrophysiological measurements

Motor and sensory nerve conduction velocities and amplitudes were not affected during the observation period (Table 2). However, mild pathological findings were recorded in some of the patients at the first examination; that is, three patients had lower than normal neCMAP, and one patient with diabetes mellitus (M6) had a sensory neuropathy (low sural nerve action potential amplitudes and absent nervus fibularis superficialis responses). Further, two patients developed a mild conduction block of the nervus fibularis at the fibula head region at the end of the observation period. On the final day of the intervention, no pathological spontaneous electromyography activity had occurred in any of the legs. The neCMAP:dmCMAP ratio was normal (>0.75) in all fully sedated and mechanically ventilated patients, except in two patients with conduction block where the ratios were 0.25 and 0.26.

**Table 2:** Electrophysiological observations

	Day 1			Day 9 ± 1		
	
	Unloaded leg	Loaded leg	*P *value	Unloaded leg	Loaded leg	*P *value
**Motor**						
Fibularis						
Amplitude (mV)	4.7 ± 0.8	4.1 ± 0.6	NS	4.0 ± 1.1	3.6 ± 0.7	NS
CV (m/second)	45.8 ± 2.5	46.6 ± 1.9	NS	45.6 ± 2.9	42.6 ± 2.2	NS
Latency (milliseconds)	4.5 ± 0.3	4.5 ± 0.4	NS	4.0 ± 0.3	4.6 ± 0.5	NS
F-response (milliseconds)	37.0 ± 3.4	26.4 ± 9.4	NS	32.1 ± 10.2	42.9 ± 8.7	NS
Tibialis						
Amplitude (mV)	7.6 ± 0.9	7.8 ± 0.9	NS	9.6 ± 1.5	9.5 ± 1.4	NS
CV (m/second)	44.7 ± 1.0	47.3 ± 2.5	NS	44.4 ± 1.6	45.6 ± 2.2	NS
Latency (milliseconds)	4.0 ± 0.3	4.4 ± 0.3	NS	3.7 ± 0.2	4.0 ± 0.3	NS
F-response (milliseconds)	49.2 ± 1.6	50 ± 1.4	NS	49.1 ± 1.8	42.5 ± 8.6	NS
**Sensory**						
Suralis						
Amplitude (mV)	11.7 ± 3.5	8.3 ± 1.6	NS	10.8 ± 2.9	8.3 ± 1.3	NS
CV (m/second)	51.9 ± 3.4	49.7 ± 3.3	NS	50.8 ± 2.1	38.4 ± 8.2	NS
Fiburalis superficialis						
Amplitude (mV)	4.6 ± 1.3	6.1 ± 1.6	NS	6.5 ± 2.9	7.0 ± 1.8	NS
CV (m/second)	40.5 ± 7.2	40.6 ± 7.1	NS	45.5 ± 8.6	40.6 ± 7.0	NS
**DMS (neCMAP:dmCMAP ratio)**				1.1 ± 0.1	1.4 ± 0.3	NS

Data presented as mean ± standard error of the mean. CV, coefficient of variation; dmCMAP, compound muscle action potential upon direct muscle stimulation; DMS, direct muscle stimulation; neCMAP, compound muscle action potential upon nerve stimulation; NS, not significant.

### Single muscle fiber properties

The CSA and force generation capacity (maximum force normalized to muscle fiber CSA; that is, specific force) were measured in single muscle fibers obtained from TA muscle biopsies from both the loaded and unloaded sides on the final day of the observation period. The TA muscle in humans is dominated by fibers expressing the type I MyHC isoform, and type IIa MyHC fibers were scarce and not observed in all patients. Analyses have therefore been restricted to muscle fibers expressing the β/slow (type I) MyHC isoform.

**Figure 2:**
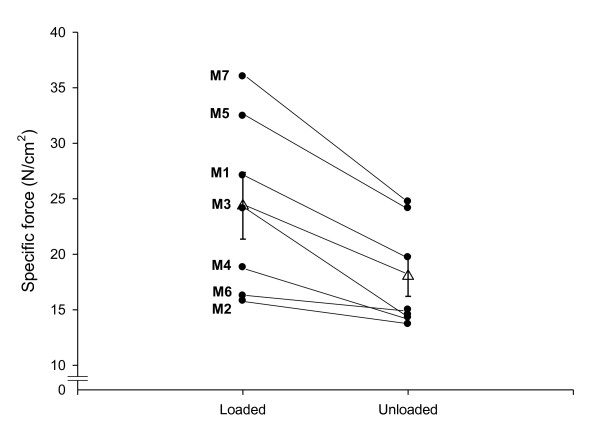
**Specific force in single muscle fibers**. Specific force in single muscle fibers expressing the type I myosin heavy chain isoform in the loaded and unloaded legs from patients exposed to unilateral loading and mechanical ventilation for 9 ± 1 days. Black circles, individual means; open triangles, average for all patients pooled together ± standard error of the mean.

A total of 140 TA muscle fibers passed the acceptance criteria (see Materials and methods) and were included in the analyses. The specific force was higher (*P *< 0.05 to 0.001) in muscle fibers on the loaded side than on the unloaded side in five out of the seven patients (Figure 2). A similar trend was observed in the other two patients (M2 and M6), but failed to reach statistical significance. Overall, 9 ± 1 days of passive mechanical loading resulted in a 35% higher specific force (*P *< 0.001) compared with the unloaded leg. The CSA measured at a fixed sarcomere length in single muscle fibers expressing the type I MyHC isoform did not differ between the loaded side (2,260 ± 140 μm^2^) and the unloaded side (2,250 ± 120 μm^2^). Similar results were obtained from morphometrical measurements of NADH-stained muscle TA muscle cross-sections; that is, the size of the slow-oxidative (type I) muscle fibers did not differ between the loaded leg (3,570 ± 90 μm^2^) and the unloaded leg (3,640 ± 100 μm^2^). Muscle fiber size measurements are thus in agreement with ultrasound measurements demonstrating similar TA muscle size on the loaded and unloaded sides.

### Myosin:actin protein ratio

**Figure 3:**
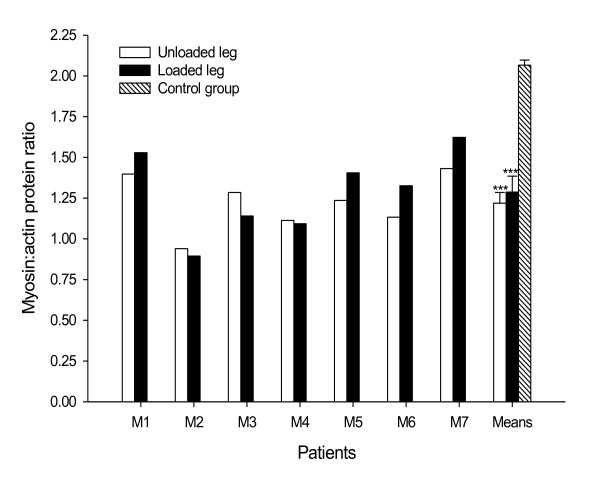
**Myosin:actin protein ratios in unloaded and loaded legs**. Myosin:actin protein ratios in tibialis anterior muscle cross-sections from each mechanically ventilated ICU patient in the unloaded leg (white bars) and the loaded leg (black bars), and from healthy controls (hashed bar). Mean ± standard error of the mean given for patients in the loaded and unloaded legs as well as in healthy controls. ***Statistically significant differences versus the healthy control group (*P *< 0.001).

In both the unloaded and loaded legs, the myosin:actin ratio was significantly lower (*P *< 0.001) in patients compared with muscle biopsies from healthy control individuals (Figure 3). Although there was a slight tendency towards a higher myosin:actin ratio on the loaded side, this difference was not statistically significant.

### Post-translational myosin modifications

A mass spectrometry approach was taken to determine myosin PTMs in response to the ICU condition *per se *as well as the effect of the passive mechanical loading. Type I and type IIa MyHC isoforms were separated on 6% SDS-PAGE gels extracted and screened for acetylation, carboxylation, deamidation, glucosylation, methylation, nitration, ubiquitination, and phosphorylation by liquid chromatography-mass spectrometry.

**Figure 4:**
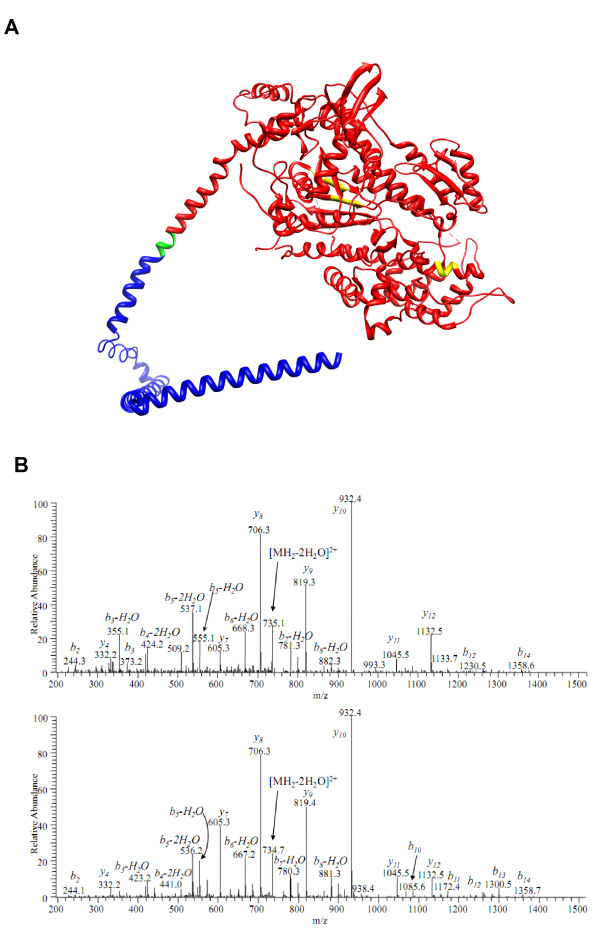
**Post-translational modifications of the myosin motor domain**. **(A) **Ribbon diagram of the myosin motor domain (red) and part of the tail region (blue). Three ICU-condition-specific post-translational modifications (PTMs) were observed in the motor domain of the protein (yellow) and one lost modification was in the tail region (green). Since only part of the myosin protein is modeled, PTMs located further down on the tail region are not shown. **(B) **Upper panel, spectrum of deamidated peptide ENQSILITGESGAGK; bottom panel, spectrum of intact peptide ENQSILITGESGAGK. The deamidation is safely determined by high-resolution mass spectrometry (MS) in MS mode (the mass difference between the molecular ion masses is 0.984 Da, corresponding to N+H-O) and in MS/MS mode as well. In the MS/MS spectrum one can see the mass shift of +1 Da for all b-ion series, including those that correspond to neutral losses from b-ions, except b_2_. Since b_2 _ion has the same mass for both peptides and contains asparagine, the deamidation is assigned to glutamine Gln3 and not Asn2.

Myosin modifications specific for the ICU condition were observed when comparing patients' samples with healthy controls (Table 3). Four modifications not observed in the controls were detected in the ICU patients; two of these modifications were observed in all seven patients and two PTMs were observed in six of the seven patients. Three PTMs were identified in the motor domain (deamidation amino acids 173 and 425, methylation amino acid 451) and one in the tail region (deamidation amino acid 1,271) (Figure 4). One modification was unique for the type I MyHC isoform (deamidation amino acid 1,271) and the other three modifications were observed in both type I and type IIa myosin isoforms. Further, five modifications were lost in the ICU patients, and all were in the tail region (acetylation amino acid 1,686 and carbonylation amino acids 780, 781, 992 and 1,427). No specific myosin PTMs were observed in response to the passive loading.

**Table 3:** Post-translational modifications specific to ICU patients

Modification type	Peptide sequence	Amino acid modified	Frequency	Position
New				
Deamidation	EN**Q**SILITGESGAGK	Q	12/14	173
Deamidation	SV**N**DLTSQR	N	10/14	1,271
Deamidation	GQTVEQVS**N**AVGALAK	N	11/14	425
Methylation	INQTL**D**TK	D	10/14	451
Lost				
Acetylation	A**S**LLAAELEELR	S	2/14	1,686
Carbonylation	AGLLGLLEEM**R**DER	R	0/14	780
Carbonylation	AGLLGLLEEMR**D**ER	D	1/14	781
Carbonylation	LQNEIE**D**LMVDVER	D	0/14	1,427
Carbonylation	NLTEEMAGL**D**ETIAK	D	1/14	992

### Neuronal nitric oxide synthase expression

**Figure 5:**
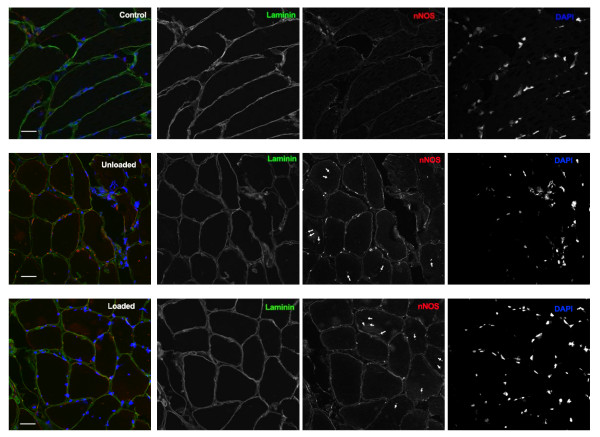
**Cross-sections of tibialis anterior muscle stained for **neuronal nitric oxide synthase**, Laminin and 4',6-diamidino-2-phenylindol**. Cross-sections of tibialis anterior muscle stained for neuronal nitric oxide synthase (nNOS; red), Laminin (green) and 4',6-diamidino-2-phenylindol (DAPI; blue). In control, basal expression of nNOS is low and localized to the sarcolemma. ICU conditions induce strong expression of nNOS and dislocation of nNOS to the cytoplasm (arrowheads). Scale bar = 50 μm. The overlay image for each row is shown to the left.

The expression of nNOS was analyzed by immunocytochemistry and basal expression was low and restricted to the sarcolemma in controls (Figure 5). In the ICU patients, a significantly higher nNOS expression was observed in the sarcolemma region (Figure 5). In addition, nNOS shuttling from the sarcolemma to the cytoplasm was observed at the end of the observation period in all ICU patients. A similar nNOS upregulation and shuttling has been reported in muscle atrophy in response muscle unloading during hind limb suspension [25] and in patients with amyotrophic lateral sclerosis [26]. No differences in nNOS expression or localization were observed in response to passive loading (Figure 5).

### Quantitative real-time PCR

**Figure 6:**
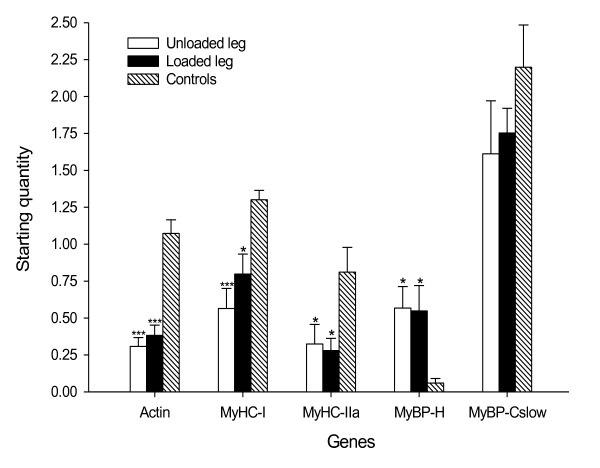
**Myofibrillar mRNA expression**. Actin, myosin heavy chain (MyHC; types I and IIa), myosin binding protein (MyBP)-H, and MyBP-C_slow _mRNA expression in the unloaded leg (white bar) and the loaded leg (black bar) from ICU patients, and in the tibialis anterior muscles from healthy controls (hashed bar). Values are starting quantity ± standard error of the mean. Statistical significance versus healthy control group: *P < 0.05 and ***P < 0.001.

Long-term immobilization and mechanical ventilation resulted in a dramatic downregulation (*P *< 0.05 to 0.001) of the dominating thick and thin filament proteins in the human TA; that is, type I and type IIa MyHCs and actin (Figure 6). Expression of the most abundant thick filament protein in the TA muscle after myosin, the slow isoform of MyBP-C, was lower but not statistically significant, and the MyBP-H expression was higher (*P *< 0.05) in the ICU patients compared with healthy controls. The expression of these genes did not differ significantly between the loaded leg and the unloaded leg (Figure 6).

## Discussion

The results from this study show that 7 to 11 days of the ICU condition resulted in a muscle phenotype suggested to be pathognomonic of the severe acquired myopathy (acute quadriplegic myopathy or CIM) observed in ICU patients; that is, a preferential myosin loss. Further, this phenotype was observed in the absence of triggering factors suggested to play an important role in the development of this myopathy, such as systemic corticosteroid hormone administration, sepsis and neuromuscular blockade. The mechanical silencing is accordingly suggested to be an important factor triggering CIM in ICU patients with or without other triggering factors such as sepsis and systemic corticosteroid hormone treatment. Furthermore, passive mechanical loading applied for 2.5 hours four times per day for 9 ± 1 days in immobilized, sedated and mechanically ventilated ICU patients improved the muscle fiber function by 35% without affecting muscle mass. These results demonstrate an important beneficial effect of passive loading on muscle function and strongly support active physical therapy as an important early intervention strategy in immobilized ICU patients in spite of the fact that it did not alleviate the muscle wasting associated with the ICU condition; that is, mechanical ventilation, sedation and immobilization.

### Preferential myosin loss

CIM is a common acquired myopathy in ICU patients and up to 42% of the ICU population may develop CIM [27]. Awareness of this condition has increased significantly in the past decade [28], but many patients with CIM still fail to receive a correct diagnosis and are being misdiagnosed as neuropathies. There is no specific treatment to date besides reducing or eliminating causative factors, and future intervention strategies need to be specific for the underlying mechanism. The poor understanding of basic mechanisms underlying CIM in the clinical setting is in part due to the basic distinctions between acquired myopathy and neuropathy often not being clearly made. Diagnosis and classification have frequently been based on clinical observations and electrophysiological measurements, but both are weak diagnostic indicators [6,29]. However, correct distinction between myopathy and neuropathy in the ICU is very important because prognosis differs significantly between the acquired neuropathy and myopathy.

A preferential loss of myosin and myosin-associated proteins has been repeatedly documented in patients with CIM using electron microscopy, electrophoretic separation of myofibrillar proteins, enzyme-cytochemistry and immuno-cytochemistry [6,30-32]. Widespread myosin loss is therefore considered to be essentially pathognomonic of CIM although myosin loss has been reported in other disorders, such as dermatomyositis [33] and cancer cachexia [34]. During the past 15 years we have routinely measured the proportion of myosin in relation to actin in 10 μm percutaneous muscle biopsy cross-sections together with electrophysiological methods in the diagnosis of acquired myopathy and neuropathy in ICU patients [5,29]. In our experience, the myosin:actin ratio is the most sensitive diagnostic tool available to detect CIM in ICU patients, being superior to electrophysiological methods or electron microscopic, enzyme-histochemical and immunocytochemical analyses of muscle biopsy cross-sections [6]. In this study, all mechanically ventilated, sedated and immobilized ICU patients had lower than normal myosin:actin ratios. This is consistent with our previous studies using a rat experimental ICU model; that is, a preferential myosin loss in both fast-twitch or slow-twitch muscles and fiber types was observed in response to mechanical ventilation, sedation and immobilization at durations longer than 5 days [8,35,36].

Systemic corticosteroid hormone treatment, post-synaptic neuromuscular blockade and sepsis have all been suggested to be important factors triggering CIM [33,37], although mechanically ventilated and sedated ICU patients have been reported with CIM in the absence of exposure to these triggering agents [38-41]. Results from this and previous experimental studies support the strong impact of mechanical silencing in triggering CIM; that is, a lack of both external (weight-bearing) and internal load (strain caused by myosin-actin activation during contraction) in mechanically ventilated and sedated ICU patients with or without neuromuscular blockade. However, the loading induced by the passive ankle joint flexion extensions for 10 hours per day was not sufficient to reduce the unloading-induced preferential myosin loss.

The mechanisms underlying the preferential myosin loss are complex and involve the activation of different proteolytic pathways in a specific temporal sequence [8,42]. However, the myosin loss is not only caused by enhanced degradation but also by decreased synthesis, as indicated by a significant downregulation of contractile proteins at the transcriptional level. This is consistent with previous observations in patients with CIM and in experimental ICU models [5,6,8,35,36]. The sparing of the thin filament protein actin in spite of a similar downregulation at the transcriptional level has been suggested to be secondary to differences in protein turnover rate or the upregulation of the small αB-crystalline chaperone protecting actin from degradation [8]. In contrast to other thick filament proteins, MyBP-H was upregulated at the transcriptional level in accordance with previous observations at the mRNA and protein levels in patients with CIM as well as in experimental ICU models [5,8,36]. The role of MyBP-H in the organization of myosin in the thick filament during myofibrillogenesis [43] and for substituting mutant MyBP-C in cardiomyopathy [44] suggest that the upregulation of MyBP-H may represent a compensatory mechanism aiming at maintaining thick filament integrity.

A mass-spectrometry approach was taken to examine the effects of the ICU condition on PTMs of myosin. A series of myosin PTMs, not identified in healthy control subjects, was observed in response to the ICU condition. In general, new PTMs were mainly observed in the motor domain while lost PTMs were detected in the tail region. Furthermore, the new PTMs were located deep within the head domain, in regions not readily accessible for oxidative modifications, and thus suggest the presence of acute oxidative stress. Oxidative stress-induced modifications of muscle proteins can result in unfolding of the targeted protein domains [45], leading to an increased exposure of hydrophobic residues that are prone to activate the proteasome [45,46].

Nitric oxide synthase is hypothesized to play a role in the modifications of the motor domains since three out of the four new PTMs were deamidations. The nitrite anion induces protein deamidation [47] and is a metabolite of the physiological messenger nitric oxide, a product of nitric oxide synthase activity. During oxidative stress observed in critically ill ICU patients [48-50], nitric oxide serves as a superoxide radical scavenger (O_2_^-^) and forms peroxynitrite (ONO_2_^−^). Peroxynitrite is a reactive oxidant and nitrating agent that is tightly regulated under physiological conditions, but it also has detrimental effects under acute oxidative stress [51]. To test our hypothesis, the expression and localization of nNOS was studied in TA muscle cross-sections from the ICU patients and healthy controls. An increased nNOS expression and a translocation from the sarcolemma to the cytoplasm were observed in all ICU patients, suggesting higher nitric oxide levels. Hind-limb suspension experiments have shown that nitric oxide levels are tied to nNOS [25], indicating that nNOS is tightly associated with elevated levels of nitric oxide in skeletal muscles. Furthermore, cytoplasmic nNOS localization leads to nitric oxide production and is a key component of the mechanism underlying disuse muscle atrophy [25]. The myosin deamidations are thus suggested to be linked to the CIM-induced change in nNOS expression and localization.

### Muscle mass and function

A linear decline in TA muscle CSA was observed during the observation period, resulting in a 22% smaller CSA at the end of the observation period. The early and linear decline in muscle mass in response to muscle unloading is different from our previous experimental findings from both porcine and rodent experimental ICU models. In the experimental studies, muscle mass and muscle fiber size were maintained during the initial 5 days and were followed by rapid muscle wasting [8,52-54]. This discrepancy may, at least in part, be explained by background heterogeneities in patients compared with animal models, the delayed monitoring of muscle mass and age-related differences in muscle wasting. In the patients, muscle mass measurements were not initiated on the first day of the ICU condition (as in the experimental studies) because of a delay in obtaining written informed consent from close relatives, and all animals in the experimental studies were young while the age of patients ranged between 56 and 67 years.

A similar average decline in muscle and muscle fiber size was observed in the loaded and unloaded legs; that is, TA muscle CSA determined by ultrasound at three different anatomical landmarks, or muscle fiber CSA measured at a fixed sarcomere length in single muscle fiber segments or from enzyme-histochemically stained muscle cross-sections. The fluid shifts reported in ICU patients [55-57] therefore appear less likely to have masked a loading effect on TA muscle mass. The significant loading effect on muscle fiber size reported by Griffiths and coworkers in five mechanically ventilated ICU patients exposed to unilateral loading of ankle joint flexors-extensors for 9 hours per day for 7 days [14] was not observed in this study. In accordance with Griffiths and coworkers, there was a large individual variability in the loading response (Figure 1B) and a maintained muscle fiber CSA was only observed in the three most critically ill patients of the five patients in their study, whereas in this study none of the patients were severely ill. Interestingly, Griffiths and coworkers used the ratio of protein to DNA as an index of muscle wasting, and they found that it decreased similarly in both limbs, concluding that the effects of passive stretching on prevention of muscle wasting remain uncertain [14]. Moreover, all patients in the study by Griffiths and coworkers required complete neuromuscular blockade, and it cannot be ruled out that neuromuscular blockade may facilitate the impact of the loading intervention. This discrepancy is supported by results from the experimental rat ICU model where unilateral passive mechanical loading in neuromuscular blockade, sedated and mechanically ventilated animals resulted in a significant muscle mass sparing effect (Renaud G, Llano-Diez M, Ravara B, Gorza L, Feng HZ, Jin JP, Cacciani N, Gustafson AM, Ochala J, Corpeño R, Hedström Y, Ford GC, Nair KS, Larsson L. 2012. Sparing of muscle mass and function by passive loading in an experimental intensive care unit model. J. Physiol (Lond.). Provisionally accepted).

Results from the porcine ICU model, on the contrary, have shown identical effects on muscle mass and function in pigs exposed to sedation and mechanical ventilation alone or in combination with neuromuscular blockade [58].

In contrast to the effects on muscle size, a consistent positive loading-induced effect was observed on muscle fiber force-generation capacity; that is, the specific force was 35% higher on the loaded side than on the unloaded side. We have made similar observations in an experimental rat ICU model where functional capacity was approximately double on the loaded side versus the unloaded side after 14 days of unilateral passive ankle joint flexions-extensions for 12 hours per day (Renaud G, Llano-Diez M, Ravara B, Gorza L, Feng HZ, Jin JP, Cacciani N, Gustafson AM, Ochala J, Corpeño R, Hedström Y, Ford GC, Nair KS, Larsson L. 2012. Sparing of muscle mass and function by passive loading in an experimental intensive care unit model. J. Physiol (Lond.). Provisionally accepted).

In both the present clinical study and the experimental study, a loading-related increase in specific force was observed despite a lower than normal myosin:actin ratio. The mechanisms underlying this unexpected finding are not known, but are speculated to be secondary to a loading-induced effect on the intrinsic properties of the contractile machinery. Mass-spectrometry analyses of myosin PTMs did not, however, show any significant differences between loaded and unloaded muscles. This investigation targeted myosin PTMs such as acetylation, carboxylation, deamidation, glucosylation, methylation, ubiquitination, and phosphorylation. Other PTMs as well as modifications of other contractile proteins, including thin filament proteins, undetected in this study may be affected by the loading condition with important consequences for regulation of muscle contraction [59]. There is a slow turnover of myosin in skeletal muscle and one cannot exclude that the initial preferential myosin loss targets a pool of newly synthesized myosin or myosin destined for degradation (that is, myosin not involved in force generation).

### Electrophysiology

Motor and sensory conduction velocities, amplitude responses and neCMAP/dmCMAP ratios were typically within the normal range and did not change significantly during the observation period. The muscle CIM phenotype observed in these patients at the end of the observation period, such as a preferential myosin loss, downregulation of contractile proteins, and upregulation of MyBP-H, was not accompanied by a significant change in these electrophysiological parameters. The sensitivity of these techniques in the early detection of CIM may accordingly be questioned. Electrophysiological methods are typically more sensitive in detecting neuropathies than myopathies and there were no findings indicating an acquired neuropathy during the observation period. We have previously shown that neuropathy does not induce a preferential myosin loss [6].

## Conclusion

A series of novel findings observed in this study are of importance for our understanding of the mechanisms underlying the acquired myopathy in ICU patients and how it is affected by passive mechanical loading. To our knowledge, none of these findings have previously been reported in ICU patients. First, the mechanical silencing unique for immobilized and mechanically ventilated ICU patients under deep sedation with or without neuromuscular blockade is forwarded as an important factor underlying the preferential myosin loss; that is, a laboratory finding considered pathognomonic for CIM in ICU patients. Second, a series of specific PTMs was observed in the motor domain of myosin that may be critical for both function and for triggering proteolysis. Furthermore, the higher nNOS expression found in the ICU patients and its cytoplasmic translocation are probably key factors in triggering these modifications. Third, passive mechanical loading had a significant and consistent positive effect on the force-generating capacity of individual muscle fibers in the loaded leg, strongly supporting the importance of early physical therapy and mobilization in deeply sedated and mechanically ventilated ICU patients.

## Key messages

• Mechanical silencing is a primary mechanism triggering the muscle weakness and the preferential myosin loss associated with CIM.

• Mechanical silencing induces specific PTMs in the motor domain of myosin.

• Increased nNOS expression as well as its dislocation to the cytoplasm is observed in ICU patients in response to mechanical silencing; moreover, they are forwarded as a probable mechanism underlying the highly specific myosin PTMs.

• Passive mechanical loading has a beneficial effect on skeletal muscle function in sedated and mechanically ventilated ICU patients.

## Abbreviations

CIM: critical illness myopathy; CMAP: compound muscle action potential; CSA: cross-sectional area; dmCMAP: compound muscle action potential upon direct muscle stimulation; HPLC: high-performance liquid chromatography; MyBP: myosin binding protein; MyHC: myosin heavy chain; nNOS: neuronal nitric oxide synthase; neCMAP: compound muscle action potential upon nerve stimulation; PCR: polymerase chain reaction; PTM: post-translational modification; RT: reverse transcriptase; TA: tibialis anterior.

## Competing interests

The authors declare that they have no competing interests.

## Authors' contributions

ML-D carried out the ultrasound measurements and analysis, compiled patients' information, participated in the enzyme histochemistry and immunocytochemistry, performed RNA extraction and quantitative RT-PCR tests, participated in the design and coordination of the study, and drafted the manuscript. GR carried out contractile measurements of single muscle fibers, performed the enzyme histochemistry and immunocytochemistry, interpreted the PTMs, participated in the design of the study, and drafted the manuscript. MA coordinated the mechanical loading intervention and compiled patient information. HGM performed the electrophysiological measurements and analysis. NC participated in the ultrasound measurements. HE recruited the patients. RC determined the myosin:actin protein ratios. KA performed liquid chromatography-mass spectrometry analysis and peptide identification. JB critically revised the PTMs. LL conceived of the study, participated in its design and coordination, and helped to draft the manuscript. All authors read and approved the final manuscript.

## Supplementary Material

Additional file 1 **a text file presenting detailed methods**. Complementary information is given about electrophysiological measurements, post-translational modifications and quantitative real-time PCR methods described in the paper. Click here for file
