# Supplementary material for: Mechanisms underlying ICU muscle wasting and effects of passive mechanical loading
Source: Crit Care. 2012 Oct 26;16(5):R209. doi: 10.1186/cc11841 (PMC3682313; doi:10.1186/cc11841)
Supplement: Additional file 1 — a text file presenting detailed methods. Complementary information is given about electrophysiological measurements, post-translational modifications and quantitative real-time PCR methods described in the paper. [file cc11841-S1.DOC]

**Additional file 1:**

**Electrophysiological measurements**

Motor (n. fibularis, and tibialis) and sensory nerve (n. suralis and fibularis superficialis) were measured bilaterally (Keypoint Medtronic, Skovlunde, Denmark) using surface electrode both for stimulation and recording. Studies were performed on the first and final day of the observation period in all patients and two of the seven patients were monitored every second day during the observation period (M6 and M7). Nerve conduction velocities were compared with reference values from age- and height-matched control subjects (Department of Clinical Neurophysiology, Uppsala University Hospital). The CMAP amplitudes upon supramaximal motor nerve stimulation were measured from m. extensor digitorum brevis (n.fibularis stimulation) and m. abductor hallucis (n.tibilalis stimulation). At the final day of the period, concentric needle electromyography (EMG) was performed in the m. vastus lateralis and tibialis anterior bilaterally (Keypoint Medtronic, Skovlunde, Denmark).

The CMAP amplitude was measured upon direct supramaximal m. tibialis anterior stimulation (dmCMAP) and compared with CMAP amplitude in response to supramaximal n. fibularis stimulation (neCMAP) bilaterally at the final day of the observation period. CMAP amplitudes were measured peak to peak. dmCMAP was measured using a monopolar stimulating needle electrode (28G, 30 mm, surface area 0.35 mm^2^; Alpine, BioMed Asp Instrument, Skovlunde, Denmark) that was placed in the distal third of the TA muscle as cathode. A similar needle electrode was used as anode (28G, 15 mm, surface area 0.35 mm^2^) placed 20-30 mm lateral to the cathode. A monopolar recording needle electrode (28G, 30 mm, surface area 0.35 mm^2^) was inserted 30 mm proximal to the cathode. A conventional non-polarisable gel surface electrode (Blue Sensor Tab, Ambu A/S, Ballerup, Denmark) was used as anode and placed at the knee. The stimulating and recording electrodes were inserted at a depth of ~20 mm perpendicular to the skin surface and maintained in this position by taping the looped electrode leads to the skin. The ground electrode was placed between the electrodes. Stimulation threshold was assessed from various stimulation points. Stimulation strength was slowly increased until supramaximal stimulation was achieved (from 1 to 100 mA with a stimulus duration of 0.1 msec). In the case no response was obtained even after 5 repositions of the stimulating cathode, the muscle was considered non-excitable.

neCMAP was measured in response to supramaximal stimulation of n. fibularis at the level of the fibular head and recording electrodes were kept in the same position as during the dmCMAP measurement. The neCMAP:dmCMAP amplitude ratio was calculated. Limb skin temperature was kept at >32 °C. Filter settings were 2 Hz-5 KHz and stimulus duration was 0.1 ms (1 Hz stimulation rate).

**Post-translational modifications**

*In-gel digestion*

Cross-sections from TA from ICU patients and m. vastus lateralis from controls were run on 6% SDS-PAGE gel. Gel bands corresponding to MyHC I and IIa were excised. Gel pieces were destained by multiple rinsing with 50:50 ACN:100 mM NH_4_HCO_3_ followed by reduction with 15 mM solution of dithiothreitol in 100 mM NH_4_HCO_3_ (30 min at 56°C) and alkylation with 2.1-fold of iodoacetamide (30 min in the dark at room temperature). The liquid was removed, gel pieces were washed with 100% ACN and then dried at 50 °C. Trypsin solution (25 ug/mL in 50 mM NH_4_HCO_3_) was added and the samples were first soaked at +8°C for 60 min and then digested at 37 °C overnight. After digestion, the samples were sonicated for 5 min and the liquid was collected and dried under vacuum.

*LC-MS analysis and peptide identification*

A 7-tesla LTQ-FT Ultra tandem mass spectrometer (ThermoFisher Scientific) modified with a nano electrospray ion source (ProxeonBiosystems) was used for all analyses. The samples were diluted in water/TFA (1:0.005 v/v), and peptides were separated by Agilent 1100 nanoflow system equipped with homemade 15-cm fused silica emitter packed with Reprosil-Pur C18-AQ 3 µm resin (Dr. Maisch GmbH), and coupled on-line to the mass spectrometer. Peptides were eluted with a 40-min linear gradient from 2% to 45% acetonitrile at 200 nL/min. The mass spectrometer was operated in positive ion mode and during each run it automatically switched between a high resolution (resolving power 50 000) survey mass spectrum in the FTMS cell and consecutive low resolution CID spectra of the 5 most abundant ions in the ion trap. CID was performed with helium as a collision gas (pressure 0.1 Pa), normalized collision energy was set to 30%. A dynamic exclusion for 30 seconds for 5 peptides was used as well. The acquired data (.RAW-files) were converted by in-house written program to Mascot generic format files. Peptide identification was performed using the Mascot search engine (version 2.1.3, Matrix Science) by searching the Uniprot-Swissprot database with set “Homo sapiens” taxonomy. Searches were performed with trypsin specificity and up to two missed cleavages were allowed. Mass deviation for precursor ions was set to 0.02 Da. For fragment ions the mass deviation was set to 0.7 Da.

Carbamidomethylation was chosen as a fixed modification, and the instrument setting was “ESI-FTICR”. The peptide/protein identifications were based on MudPIT scoring. To search all possible posttranslational modification the searches were repeated four times for each sample. The searches were performed with all above mentioned settings and variable modifications set to: (a) deamidation (N, Q), oxidation (M), phosphorylation (S,T), phosphorylation (Y), methylation (C-terminus), and methylation (D, E); (b) deamidation (N, Q), oxidation (M), oxidation (W), oxidation (H), oxidation (R), oxidation (P), oxidation (Y); (c) deamidation (N, Q), oxidation (M), oxidation (D), oxidation (F), oxidation (K), oxidation (N); (d) deamidation (N, Q), oxidation (M), acetylation (K), acetylation (C-terminus), acetylation (C), acetylation (S), nitration (Y), nitration (W).

The output Mascot results were exported as XML files, and data with a peptide scores below 25 were excluded. All remaining peptides we filtered by exclusion of: (a) non-modified peptides; (b) peptides containing only oxidation (M) and/or deamidation (N, Q); (c) peptides belonging to any proteins others than myosin family; (d) same peptides identified multiple times in the same sample. The myosin modeling used in the study has been described extensively elsewhere and visualized with UCSF Chimera .

**Quantitative real-time-PCR**

qRT-PCR was used to quantify the mRNA levels for human type I and IIa MyHCs, skeletal α-actin, myosin binding protein C slow (MyBP-C_slow_) and myosin binding protein H (MyBP-H; GenBank accession M58018, [AF111784](http://www.sciencedirect.com/science?_ob=RedirectURL&_method=externObjLink&_locator=genbank&_issn=0022510X&_origin=article&_zone=art_page&_plusSign=%2B&_targetURL=http%253A%252F%252Fwww.ncbi.nlm.nih.gov%252Fentrez%252Fquery.fcgi%253Fcmd%253Dsearch%2526db%253Dnucleotide%2526doptcmdl%253Dgenbank%2526term%253DAF111784%5Baccn%5D), NM_001100, NM_002465, NM_004997, respectively).

100 ng of total RNA from TA samples were reverse transcribed to cDNA using Qscript cDNA supermix (Quanta Biosciences, USA). cDNA was amplified in triplicate using MyiQ™single color real time PCR detection system (Bio-Rad Laboratories, Inc., Hercules, CA, USA). The thermal cycling conditions include 95 °C for 10 minutes, followed by 50 cycles of a two-step PCR with denaturation at 95 °C for 15 seconds and a combined annealing and extension step at 60 °C for 1 minute. SYBR Green (1988123, Roche Diagnostics GmbH, Germany) was used as DNA-binding fluorophore. Each reaction was performed in a 25µl volume with 0.4µM of each primer and 0.2µM of SYBR Green. When optimising each PCR, the PCR products were run on 2% agarose gels to ensure that primer-dimer formation was not occurring. Taqman primers were designed using the software Primer Express® (Applied Biosystems, Foster City, CA, USA). Primer sequences have been published elsewhere and were purchased from Thermo Electron (Thermo Electron, Ulm, Germany). All primers were purified by high-performance liquid chromatography. Threshold cycle (Ct) data obtained from running qRT-PCR was related to a standard curve to obtain the starting quantity (SQ) of the template cDNA. The values were normalized against 28S (GenBank accession M11167).

**References**

1. Wendt, T., Taylor, D., Trybus, K.M., and Taylor, K. 2001. Three-dimensional image reconstruction of dephosphorylated smooth muscle heavy meromyosin reveals asymmetry in the interaction between myosin heads and placement of subfragment 2. *Proc Natl Acad Sci U S A* 98:4361-4366.

2. Pettersen, E.F., Goddard, T.D., Huang, C.C., Couch, G.S., Greenblatt, D.M., Meng, E.C., and Ferrin, T.E. 2004. UCSF Chimera--a visualization system for exploratory research and analysis. *J Comput Chem* 25:1605-1612.

3. Norman, H., Andersson, P., Nordqvist, J., Zackrisson, H., Larsson, L. 2009. Changes in myofibrillar protein and mRNA expression in patients with Acute Quadriplegic Myopathy during recovery. *Journal of the Neurological Sciences* In press.
